# Multigenerational endometriosis : consequence of fetal exposure to diethylstilbestrol ?

**DOI:** 10.1186/s12940-021-00780-5

**Published:** 2021-08-27

**Authors:** Laura Gaspari, Marie-Odile Soyer-Gobillard, Françoise Paris, Nicolas Kalfa, Samir Hamamah, Charles Sultan

**Affiliations:** 1 CHU Montpellier, Univ Montpellier, Unité d’Endocrinologie-Gynécologie Pédiatrique, Service de Pédiatrie, Montpellier, France; 2 CHU Montpellier, Univ Montpellier, Centre de Référence Maladies Rares du Développement Génital, Constitutif Sud, Hôpital Lapeyronie, Montpellier, France; 3 Univ Montpellier, INSERM 1203, Développement Embryonnaire Fertilité Environnement, Montpellier, France; 4 CNRS, Université Sorbonne, Paris, France; 5 Association Hhorages-France, F-Asnières-sur-Oise, France; 6 CHU Montpellier, Univ Montpellier, Département de Chirurgie Pédiatrique, Hôpital Lapeyronie, Montpellier, France; 7 CHU Montpellier, Univ Montpellier, Département de Biologie de la Reproduction, Biologie de la Reproduction/DPI et CECOS, Montpellier, France

**Keywords:** Familial endometriosis, Diethylstilbestrol (DES), Multigenerational transmission, Prenatal exposure, Endocrine-disrupting chemicals (EDC)

## Abstract

**Background:**

Endometriosis, which affects 10–15 % of women of reproductive age, is an estrogen-driven condition influenced by environmental and genetic factors. Exposition to estrogen-like endocrine-disrupting chemicals (EDCs) has been reported to contribute to the fetal origin of this disease.

**Case presentation:**

We report here an informative family in which all prenatally DES-exposed daughters and subsequent granddaughters presented endometriosis, whereas the unexposed first daughter and her progeny presented no gynecological disorders. Moreover, the only post-pubertal great-granddaughter, who presents chronic dysmenorrhea that remains resistant to conventional therapy, is at risk of developing endometriosis. The mother (I-2) was prescribed DES (30 mg/day for 3 months) to inhibit lactation after each delivery.

**Conclusions:**

Although a direct causal link between the grandmother’s treatment with DES and the development of endometriosis in possibly three exposed generations remains speculative, this report strengthens the suspicion that fetal exposition to DES contributes to the pathogenesis of adult diseases, such as endometriosis. It also highlights a multigenerational and likely transgenerational effect of EDCs.

## Background

Endometriosis is an estrogen-driven condition characterized by the presence of endometrial-like tissue outside the uterus, most commonly engrafted within the peritoneal cavity secondary to retrograde menstruation. It affects 10 to 15 % of the women of reproductive age, often resulting in severe, chronic pelvic pain, dyspareunia and altered fertility [1].

The pathophysiology of endometriosis stems from a broad spectrum of genetic factors and environmental influences [2]. Retrograde menstruation is influenced by a genetic predisposition and is involved in inflammation, angiogenesis and vascularization processes [3, 4]. Other factors such as oxidative stress, resistance to apoptosis and immunological dysregulation also contribute to this disease [4].

Environmental factors also exert a considerable impact through epigenetic mechanisms [5]. Increased estrogen activity during fetal life seems to be an important factor of endometriosis onset and progression [6], and epidemiological studies supports a higher rate of endometriosis among women exposed to diethylstilbestrol (DES) in utero [7–11]. In particular, Missmer et al. reported, in a prospective study on a population of 84,446 premenopausal women, 1,226 cases of self-reported laparoscopically-confirmed endometriosis with no past infertility and found that the rate of endometriosis was 80 % greater among women exposed to diethylstilbestrol in utero (RR 1.8, CI 1.2–2.8) [7, 11]. Upson et al. carried a population-based case–control study composed by 310 cases and 727 controls and suggested increased endometriosis risk with maternal use of DES (OR 2.0, 95 % CI 0.8–4.9), although not statistically significant [8]. Ottolina et al. performed a meta-analysis based on Missmer’s and Upson’s studies and the pooled results showed that DES significantly increased the risk of developing endometriosis (RR 1.92, 95 % CI 1.30 to 2.83), with non-significant heterogeneity among the studies (P = 0.98) [9]. Last, Wolff et al. performed a cohort study in which an operative cohort, composed by 473 women undergoing laparoscopy/laparotomy, was matched to an age- and residence-matched population cohort, comprising 127 women undergoing pelvic magnetic resonance imaging (MRI) [10]. The authors found that none of the women in the population cohort reported DES exposure, in contrast to 2 % of women in the operative cohort; however, within the cohort of women undergoing laparoscopy/laparotomy, the prevalence of prenatal DES exposure was almost identical [10]. Moreover, the link between endometriosis and fetal exposure to endocrine-disrupting chemicals (EDCs) with estrogen-like activity, such as dioxins, organochlorine pesticides, bisphenols and phthalates, has been established by several investigators [11, 12]. All together, these data suggest a developmental or in utero origin of this disease [13].

We had the opportunity to manage an informative family with endometriosis occurring in the second and third generations of a woman who took DES for cessation of breast feeding after each of her 10 pregnancies. All 6 daughters exposed in utero by DES presented endometriosis and all their female offspring did, as well.

This observation strongly supports the possible relationship between prenatal exposure to DES and subsequent development of endometriosis, as reported by other authors [7–9, 14]. In addition, it reinforces the suspicion of a multigenerational (and likely transgenerational) effect of DES and other EDCs in human diseases [15–22].

### Case presentation

**Fig. 1:**
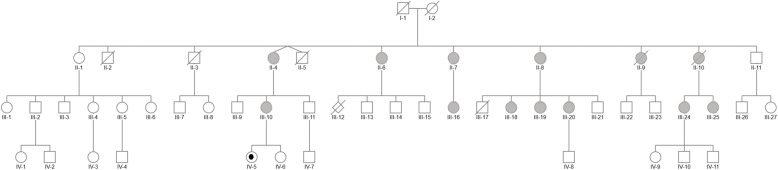
Genealogical tree of an informative family in which DES had been administered to the mother (I-2) for 3 months after the birth of each of her 11 children (4 boys + 7 girls) (II-1 to -11) to stop lactation, between 1956 and 1971. The first child (II-1) was thus not exposed to DES, since the mother started DES treatment after her birth. Six of the daughters (II-4, II-6, II-7, II-8, II-9, II-10) suffered from endometriosis (gray), whereas the oldest daughter (II-1, not exposed) and her descendants did not present this disease. The 11 children gave birth to 26 grandchildren (13 boys and 13 girls) (III-1 to -27), of which III-10, III-16, III-18, III-20, III-24 and III-25 have endometriosis. The fourth generation (IV-1 to -11) is beginning, and IV-5 is the only one to have attained menarche, with severe, chronic drug-resistant dysmenorrhea, possibly hiding endometriosis (gray point). We also note that III-11 and III-13 presented hypospadias

We report on a single family of Caucasian origin with 2 (and likely 3) generations affected by endometriosis (see pedigree, Fig. 1). The index case (II-7) joined the French association of DES-treated women (HHORAGES Association), reporting laparoscopically confirmed endometriosis. The family history revealed other family members with the same disease.

The mother (I-2) from Canada presented no symptoms of endometriosis, nor other gynecological disorders. She had worked as a waitress and then as a nurse until retirement. The father (I-1) had been a miller and then a primary school teacher until retirement. They had 11 full-term children (one twin pregnancy). The mother (I-2) was thus gravida/para/abortion (GPA) G10 P11 A0. She underwent surgery (3 times) for recurrent benign breast tumors. She also presented severe Basedow disease and died at 80 years old from complications of vascular dementia. The mother (I-2) had 2 half-sisters and the father (I-1) had 3 sisters. No one in the extended family presented symptoms of endometriosis, infertility or breast or genital cancers.

DES (30 mg/day PO) was systematically prescribed, by the same gynecologist, to the mother for voluntary suppression of puerperal lactation for aesthetic reasons for 3 months after each delivery, starting from the first one. The medication adherence was complete. The first child (II-1) was thus not exposed to DES, since the mother started DES treatment after her birth. The first child (II-1) was a daughter and presented no symptoms of endometriosis or infertility. All the following daughters of the second generation (II-4, II-6, II-7, II-8, II-9, II-10) presented chronic dysmenorrhea treated with analgesic drugs and oral contraceptive pills (OCP) and had laparoscopically diagnosed endometriosis. In addition, II-6, II-7 and II-9 presented repetitive miscarriages, II-6 aborted an anencephalic fetus (III-12), and II-8 gave birth to 2 very premature children; the first died at 3 days (III-17) and the second (III-20) survived.

Regarding the third generation, all the females born to the affected daughters of the second generation (III-10, III-16, III-18, III-19, III-20, III-24, III-25) presented chronic dysmenorrhea treated with painkillers and OCP and were noninvasively diagnosed with endometriosis (transvaginal ultrasound and/or magnetic resonance imaging: MRI). It should be noted that none of the daughters born to the only unexposed female (II-1) presented symptoms of endometriosis.

In the fourth generation, IV-5 is the only great-grandchild who has attained menarche. She is now 15 years old and since menarche has presented severe, chronic dysmenorrhea, resistant to treatment.

Interestingly, III-11 and III-13 presented hyspospadias.

The extended family presented no occupational or environmental exposure to EDCs, except for III-25 (aesthetician).

The family members, now spread over Europe and Canada, declined to offer permission for molecular investigations.

## Discussion

We report here an informative family in which all prenatally DES-exposed daughters and subsequent granddaughters presented endometriosis, whereas the unexposed first daughter and her progeny presented no gynecological disorders. To our knowledge, this familial expression of endometriosis in 2 generations, and likely 3 generations, associated with DES exposure has never been reported.

The mother (I-2) was systematically prescribed DES (30 mg/day PO for 3 months) to inhibit lactation after each delivery. The frequency of DES treatment prescribed to stop lactation after delivery was not yet documented in the scientific literature and, to our knowledge, no study reported its impact on the development of children born after such treatment. Earlier studies on the pharmacodynamics of DES in mammals reported that 120 days after ^14^ C-DES treatment, DES concentration was measurable in the liver [23]. Since the biological disposition of DES in humans is not extensively different from that observed in laboratory animals, it is likely that the mother’s pregnancies started under DES exposure as the interpregnancy intervals, for daughters, ranged between 3 and 8 months. Whether there would be enough DES remaining in the body to affect these pregnancies, this point constitutes a potential limitation. Actually, DES is mainly metabolized to its catechol quinone, which reacts with DNA to form adducts [24] that are stored in adipose tissue. Its overall accumulation may have induced exposure that lasted up to the beginning of each new gestation after the first. Besides, quinones are considered as deleterious disruptors during development, inducing severe modifications into single or double DNA strands during their metabolization [25], genotoxic effects occurring transplacentally on exposed fetus [26]. In addition, they are known to be able to induce specific genotoxic link to DNA [24] and likely all along several generations [27], reinforcing the suspicion of a multigenerational (and likely transgenerational) transmission of endometriosis in this informative family.

Endometriosis is known to have familial components, with the total risk of endometriosis in first-degree patients’ relatives increased up to 10.2 % versus 0.7 % in controls [28]. A recent meta-analysis identified 5 loci regulating steroid hormone pathways [29], 5 transduction signals and 19 single-nucleotide polymorphisms associated with endometriosis [30]. We were unfortunately unable to collect blood DNA from this family to investigate single nucleotide gene polymorphisms. However, no mutations strongly associated with disease risk were identified in family-based or case control linkage and candidate gene studies of endometriosis [2, 31, 32].

In addition, a growing body of literature has focused on the association between endometriosis and exposure to DES and other EDCs with estrogen-like activity [6–11]. In immortalized human endometrial cell lines, Bruner et al. partially elucidated the mechanistic processes that link EDCs and endometriosis, such as steroidogenic factor 1 gene (SF-1) overexpression causing excessive production of estrogen via high levels of ER-β, progesterone resistance, oxidative stress and pro-inflammatory conditions, which are thought to be involved in the migration, adhesion and progression of endometrial tissues [33]. Moreover, DES has been reported to induce epigenetic changes: many animal studies have identified DES exposure-related epigenetic modifications, including DNA methylation changes [34–38], although the results of human studies remain partially inconclusive [39, 40].

In this family, a direct causal link between the mother’s treatment with DES and the development of endometriosis in all prenatally DES-exposed daughters and subsequent granddaughters cannot be demonstrated and thus remains speculative. Whether there would be enough DES remaining in the body to affect these pregnancies and it might be helpful to add this as a potential limitation.

Nevertheless, the absence of endometriosis in the first unexposed daughter and her progeny indirectly suggests the key role of environmental influences. Since no single genetic abnormality can predict the development of endometriosis in all exposed daughters, DES was probably the major risk factor for the endometriosis present in all 6 daughters exposed during fetal life and the 7 granddaughters born to the affected daughters, suggesting a multigenerational impact of DES. Moreover, regarding the only post-pubertal great-granddaughter, even though a diagnosis of endometriosis has not yet been confirmed, clinicians suspect that the persistent, severe, drug-resistant dysmenorrhea is actually hiding endometriosis in this adolescent. If this is confirmed, it will provide powerful support for the suspected transgenerational effect of DES and EDCs in humans.

Only a few studies have considered DES and its multigenerational outcomes [41]. We and others have reported an increased risk of hypospadias in sons of DES daughters [15–18] and, interestingly, III-11 and III-13 presented hypospadias. Titus et al. found delayed menstrual regularization, higher odds of irregular menstrual periods and amenorrhea, and an increased risk of preterm delivery and fewer live births among women whose mothers were exposed in utero to DES [19, 42], as well as a higher rate of birth defects in DES grandchildren [20]. We recently reported primary clear cell carcinoma of the cervix in an 8-year-old DES granddaughter as possible evidence of multigenerational transmission of DES [21]. In addition, Kioumourtzoglou et al. provided evidence that DES exposure was associated with multigenerational neurodevelopmental deficits, such as attention-deficit/hyperactivity disorder (ADHD) [22].

## Conclusions

This informative family highlights, for the first time, suspicion of the multigenerational and possible transgenerational effect of DES in endometriosis. It strengthens the body of work suggesting the role of fetal exposure to EDCs in the pathogenesis of adult reproductive disease.

## Data Availability

Not applicable
